# Draft Genome Sequence of the N_2_-Fixing Cyanobacterium *Nostoc piscinale* CENA21, Isolated from the Brazilian Amazon Floodplain

**DOI:** 10.1128/genomeA.00189-16

**Published:** 2016-03-31

**Authors:** Tiago Leão, Pedro Ivo Guimarães, Aline Grasielle Costa de Melo, Rommel Thiago Jucá Ramos, Pedro Nuno Leão, Artur Silva, Marli Fatima Fiore, Maria Paula Cruz Schneider

**Affiliations:** a Center for Marine Biotechnology and Biomedicine, Scripps Institution of Oceanography, University of California San Diego, La Jolla, California, USA; b Department of Biological Systems Engineering, Virginia Tech, Blacksburg, Virginia, USA; c Federal University of Pará, Institute of Biological Sciences, Center of Genomic and System Biology, Belém, Pará, Brazil; d Center for Nuclear Energy in Agriculture, University of São Paulo, Piracicaba, São Paulo, Brazil; e Interdisciplinary Centre of Marine and Environmental Research (CIIMAR/CIMAR), University of Porto, Porto, Portugal

## Abstract

We announce here the draft genome sequence of *Nostoc piscinale* CENA21, a diazotrophic heterocyst-forming cyanobacterium isolated from the Solimões River, Amazon Basin, Brazil. It consists of one circular chromosome scaffold with 11 contigs and total size of 7,094,556 bp. Secondary metabolite annotations indicate a good source for the discovery of novel natural products.

## GENOME ANNOUNCEMENT

The *Cyanobacteria* are among the most ancient life forms on Earth, dating back approximately 3 billion years ago, and they are responsible for the rise of the atmospheric oxygen on the primitive Earth (1). They are a group of photosynthetic prokaryotes capable of surviving in almost every conceivable environment. The great adaptation skills of cyanobacteria are directly related to their capacity to produce several bioactive natural compounds (2). In particular, the genus *Nostoc* is known for the production of several highly bioactive compounds, such as cryptophycins, nosperin, nostocyclopeptide, nostopeptolide, and nostophycin (3–7), as well as potent toxins, like microcystin and nodularin (8, 9). This genus is also one of the most common dinitrogen (N_2_)-fixing cyanobacteria found in terrestrial*,* aerial, and aquatic ecosystems (10). However, in contrast to several other cyanobacterial genera, the number of *Nostoc* genomes currently available remains fairly low. Therefore, we announce here the draft genome sequence of *Nostoc piscinale* CENA21, isolated from a floodplain soil of Mari-Mari Island, Solimões River, Amazon Basin, Brazil (02°45’00.00′’S, 65°15′00.00″W), in August 1985, as previously described (11), and this strain is maintained in the culture collection of CENA/USP in Piracicaba, São Paulo, Brazil. DNA extraction was performed using a classic phenol-chloroform protocol (12) optimized to cyanobacteria. Genomic DNA was sequenced by both the Ion Torrent PGM platform, using the Ion Torrent 318 Chip, and SOLiD 5500xl, using mate-pair 50-bp reads. Reads were filtered using the FASTX-toolkit, and a minimum threshold of Phred 20 to 50% of the reads’ length. The filtered reads from Ion Torrent sequencing were assembled with the MIRA version 4.0 assembler, and the contigs were curated using the Lasergene platform. The assembled contigs were binned by a pipeline adapted from Albertsen et al. (13). *Nostoc* sp. strain PCC 7107 (with the highest average nucleotide identity to CENA21 of approximately 87.5%) was selected as the reference genome and used to order the binned contigs into scaffolds by the software CONTIGuator, and then the software GapFiller was used to reduce the number of Ns and gaps. The final draft of the *N. piscinale* CENA21 genome consists of a single circular scaffold (11 contigs), total size of 7,094,556 bp, G+C content of 40.54%, and average coverage of 129×. Expert review annotation from the Joint Genome Institute (JGI) IMG/ER database confirms that this strain harbors required genes for photosynthesis, N_2_ fixation, heterocyst formation, biosynthesis of most proteinogenic amino acids, and important cofactors. According to the presence/absence of 79 single-copy genes found in 95% of all 44 *Nostocales* genomes currently at the IMG/ER database, we estimate the genome completeness of the announced strain to be approximately 96.2%. The prediction of secondary metabolites using the antiSMASH tool indicates that 8.3% of the assembled genome is dedicated to natural product biosynthesis, grouped into 16 biosynthetic gene clusters (BGCs), including 6 bacteriocins, a nonribosomal peptide-synthetase (NRPS), 3 hybrid NRPS/polyketide synthases (PKS), 3 terpenes, and a lantipeptide. Preliminary analysis indicated that most of those BGCs are cryptic, representing a good source for the discovery of novel natural products.

### Nucleotide sequence accession number.

The *N. piscinale* CENA21 strain draft genome and annotation information were deposited in DDBJ/EMBL/GenBank under the accession no. CP012036.
